# Dynamic metabolomics of uterine fluid of laying hens: Key metabolites and pathways governing sperm motility and functional longevity

**DOI:** 10.1016/j.psj.2026.107102

**Published:** 2026-05-08

**Authors:** Qi Zhang, Hongfeng Du, Caiyue Ge, Zhouying Zhang, Jingwei Yuan, Yunlei Li, Adamu Mani Isa, Jilan Chen, Yanyan Sun

**Affiliations:** a State Key Laboratory of Animal Biotech Breeding, Institute of Animal Science, Chinese Academy of Agricultural Sciences, Beijing 100193, PR China; b College of Animal Science and Technology, Qingdao Agricultural University, Qingdao 266109, Shandong, PR China; c Department of Animal Science, Usmanu Danfodiyo University, PMB 2346, Sokoto, Nigeria

**Keywords:** Chicken, Uterine fluid, L-arginine, Sperm, Metabolite

## Abstract

The uterine fluid (**UF**) in hens constitutes a critical microenvironment for sperm entering the oviduct. The variation of metabolic components in the UF of two distinct physiological phases, plumping fluid (**PF**) and calcifying fluid (**CF**), may exert different effects on sperm storage, survival, and migration within the oviduct and thus influencing fertilization success. This concerns the efficiency of artificial insemination (**AI**) and *in vitro* sperm storage strategy. Therefore, this study aims to clarify this effect, and identify the underlying key metabolic components and mechanism. The sperm incubated with PF, CF, and PBS (control) for 6 h at 4°C was checked for sperm motility and other parameters including curvilinear velocity (**VCL**), straight-line velocity (**VSL**), average path velocity (**VAP**), and amplitude of lateral head displacement (**ALH**). Non-targeted metabolomics approach was employed to profile the metabolites present in the PF and CF. The key differential metabolite was supplemented to PBS at two concentration, 0.143 mM and 0.287 mM to incubate the sperm for 6 hours before the AI to confirm its potential effect. The results demonstrated that PF significantly enhanced sperm motility and key kinetics parameters (VSL, VAP, VCL) compared to CF and PBS during *in vitro* storage. Metabolomic analysis revealed profoundly distinct metabolic landscapes between PF and CF, which were enriched in pathways central to cellular energetics and homeostasis, including the tricarboxylic acid cycle (TCA cycle), lipid metabolism, and arginine biosynthesis and metabolism. Notably, the presence of sperm within the UF induced dynamic shifts in these metabolic profiles, particularly affecting lipid and fatty acid related metabolites. Consistent with the prominent enrichment of arginine-related metabolic pathways, the supplementation of L-arginine into PBS extender significantly prolonged sperm survival *in vitro* and improved subsequent *in vivo* fertility rate following AI. This study elucidates the intricate metabolic regulatory mechanisms within chicken UF that underpin sperm storage capacity and fertilization competence. It also highlights L-arginine as a potent additive for chicken semen extenders, offering a strategy to optimize chicken reproductive efficiency.

## Introduction

As a core microenvironment for sperm migration, capacitation, and fertilization within the female reproductive tract, the oviductal fluid, composed of ions, nutrients, bioactive factors, and metabolites, regulates sperm function through multiple pathways. Oviductal fluid from a wide range of species, including cattle (Küçük et al., 2020), horse (Pinto-Bravo et al., 2025), donkeys (Catalán et al., 2025), and black rockfish (Chen et al., 2024), has been shown to enhance sperm survival, extend fertility potential, and enhance the competence of acrosome reaction.

It is unique in poultry that sperm can be stored in the sperm storage tubules (**SSTs**) located in the mucosa at the uterovaginal junction (**UVJ**) of the female reproductive tract (Birkhead and Moller, 1992; Blesbois and Brillard, 2007; Orr, 2012) and remain viable for days to weeks after a single artificial insemination (AI) or natural mating (Das et al., 2008; Khillare et al., 2018; Matsuzakia, 2024). Spermatozoa are released from the SSTs and transferred to the oviduct, where they regain motility. Subsequently, they ascend through the oviduct and ultimately fuse with the ovum.

Uterine fluid (**UF**) serves as the important fluid medium for sperm storage and migration after release in the SSTs (Gao et al., 2025). It is worth noting that throughout the laying cycle, UF display pronounced rhythmic fluctuations that are tightly synchronized with the sequential phases of eggshell calcification (Hincke et al., 2012), may also be generally regulated by the circadian clock (Li et al., 2024). During the eggshell formation process, uterine activity increases. At this time, the components of UF released may stimulate the activity of sperm stored in the spermatotheca and help with their ascent in the oviduct to the infunnelium (Burke et al., 1969; Riou et al., 2020). The uterus produce plumping fluid (**PF**) for 5 h after the arrival of a membranous egg in the uterus (the plumping phase), and produce calcifying fluid (**CF**) for 12 h after the termination of the PF secretion (calcifying phases) (Ahammad, 2013; W. H. Burke, 1972). Previous research showed that the inclusion of PF in Lake’s solution (**LS**) prolonged the life span and improved the quality of the stored spermatozoa through maintaining survivability probably by stabilizing the membrane integrity of the acrosome (Ahammad et al., 2013). Furthermore, the secretory active stage of the female reproductive tract has an impact on sperm storage efficiency (Ahammad, 2013). They indicated that the UVJ-SSTs filling rate of sperm diluted with PF was higher than that diluted with CF. It is assumed that sperm entering the female reproductive tract during the plumping phase can be stored more effectively, allowing the longer duration of fertile eggs egg production. Chicken UF composition has been widely investigated in layer hens in relation to shell biomineralization (Gautron et al., 2021). However, there is a paucity of research that identifies the exact metabolite differences of PF and CF underlying the varying effects on fertility potential. Furthermore, it is widely reported that sperm induced the significant gene expression change in the SSTs, UVJ, and uterus (Atikuzzaman et al., 2015, 2017; Das et al., 2006), it is unclear whether these alterations are associated with concomitant changes in UF metabolite profiles.

The present study was based on the hypothesis that UF contains specific molecules that are essential for sperm survival, release from the SSTs, and the ascent trough the uterus. Thus, we first employed non-targeted metabolomics technology to identify PF and CF metabolites profiles of both sperm-inseminated (Sperm) and non-inseminated (Non sperm) hens to further identify the key role of UF metabolites and pathways in maintaining sperm motility. The results may provide a theoretical basis for optimizing AI and sperm storage techniques in poultry and improving reproductive efficiency.

## Materials and methods

### Ethical consideration

All animal experiments were approved by the Animal Care and Use Committee at the IAS-CAAS (No. IAS2025-76, 2025-03-16) and conducted at the institute. All of the experiments followed relevant guide- lines and regulations set by the Ministry of Agriculture and Rural Affairs of the People's Republic of China.

### Experimental animals and UF sample preparation

In this experiment, a total of 150 White Leghorn hens and 30 roosters of 36 wk of age kept on the experimental farm at Institute of Animal Science of Chinese Academy of Agricultural Sciences (**IAS-CAAS**) were used. All birds were caged individually and were subjected to a 16-h light and 8-h dark light regimen (16L: 8D).

**Fig. 1:**
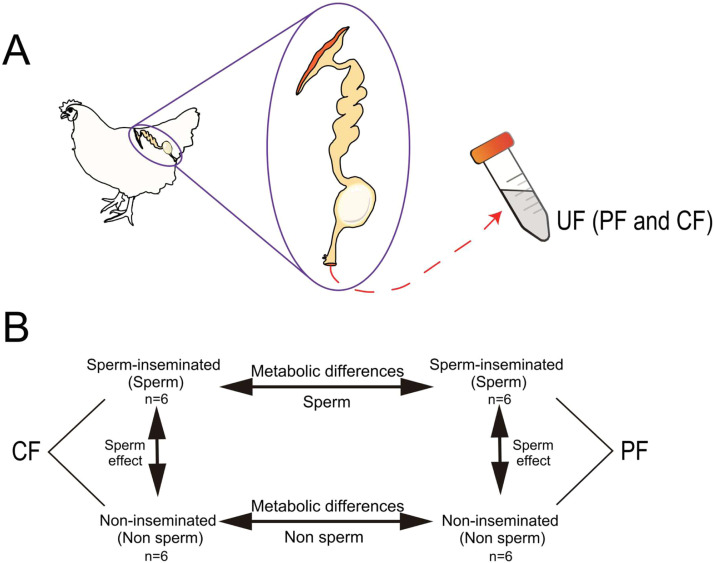
**Experimental outline. (A) Collection of uterine fluid. (B) Schematic representation of the experimental design**. Note: The UF metabolic effect between both lines was obtained by comparing both kinds of samples sperm-inseminated (Sperm) or non-inseminated (Non sperm) hens. The sperm effect within each line was obtained by comparing samples collected sperm-inseminated (Sperm) and non-inseminated (Non sperm) hens. UF, Uterine fluid; PF, plumping fluid; CF, calcifying fluid.

A number of 24 healthy hens with consistent egg-laying cycles (mean interval: 24 ± 0.5 h, confirmed via 3-day continuous monitoring for oviposition), were further selected. Hens accepted AI 24 hours ahead were used to collect PF (n = 6, CF-Sperm) at 5-8 hours post a previous oviposition and CF (n = 6, PF-Sperm) at 17-20 hours post a previous oviposition), while the hens without AI were used to collect PF(n = 6, CF-Non sperm) and CF (n = 6, PF-Non sperm) (Figure 1A) following the description in a previous study (Sun et al., 2013). In brief, the oviposition was induced by intravenous injection of prostaglandin F2A (50 µg/hen, Yuanye, S28690, China), and UF was collected using a plastic tube placed at the entrance of the everted vagina immediately after the egg expulsion. The collected UF was rapidly frozen in liquid nitrogen, and transferred to be stored at −80°C until further manipulation.

### Assessment of sperm quality for semen incubated with UF in vitro

Fresh semen collected from the 30 healthy roosters with proven sperm quality was thoroughly mixed and then equally divided into three groups. Each group included 3 replicates of 500 μL semen. The three groups were diluted at a ratio of 1:1 with PF, CF, and PBS (as a control), respectively, and stored at 4°C for the assessment of the sperm motility and other parameters including curvilinear velocity (**VCL**), straight-line velocity (**VSL**), average path velocity (**VAP**), and amplitude of lateral head displacement (**ALH**) using a computer-aided sperm analysis (**CASA**) system (IVOS II; Hamilton Thorne, Inc., Beverly, MA 01915, USA)at 0, 2, 4, 6, and 8 h, respectively. The motility assessment protocol for rooster sperm, including all CASA system configurations on the Hamilton-Thorne motility analyzer, was preconfigured by the manufacturer as detailed in Supplementary Table 1. Prior to each analysis session, manual calibration of minimum head brightness and head area parameters was performed based on microscopic field illumination to ensure accurate identification of avian spermatozoa.

### Sample preparation for liquid chromatography-mass spectrometry analysis

Frozen specimens stored at −80°C were thawed in an ice-water slurry. A 100 μL aliquot of each thawed sample was pipetted into a 1.5 mL microcentrifuge tube. Protein precipitation was then initiated by adding 400 μL of a methanol-acetonitrile mixture (2:1, v/v) containing mixed internal standards at a concentration of 4 μg/mL. The mixture was subjected to vortex oscillation for 1 min, ultrasonic extraction in an ice water bath for 10 min, and subsequent overnight incubation at −40°C. After centrifugating at 12,000 rpm and 4°C for 20 min, a total of 150 μL of the supernatant was carefully transferred into an Liquid Chromatography-Mass Spectrometry (**LC-MS**) injection vial fitted with a conical inner liner tube for subsequent analysis. Quality control (**QC**) samples were prepared by pooling equal-volume aliquots of the supernatants derived from all individual extracts.

### LC-MS analysis

LC-MS based metabolomics analysis was performed using an ultra-high-performance liquid chromatography system (Waters ACQUITY UPLC I-Class plus, Shanghai, China) coupled with a high-resolution mass spectrometer (Thermo QE HF, Shanghai, China). Chromatographic separations of UF samples (Fig. 1B) were achieved on an ACQUITY UPLC HSS T3 column (2.1 mm × 100 mm, 1.8 µm) with the column temperature maintained at 45°C and the flow rate set at 0.35 mL/min. The mobile phase comprised phase A (0.1% formic acid in aqueous solution) and phase B (pure acetonitrile), and gradient elution was performed according to the program. The injection volume of each sample was 4 µL.

### Identification of metabolites and statistical analysis of data

After obtaining the LC-MS spectrogram of 24 UF samples, the raw mass spectrometry data were processed using Progenesis QI software (Progenesis QI v3.0, Nonlinear Dynamics, Newcastle, UK) for peak identification, filtration, and, alignment. Two-dimensional matrices containing the m/z values and peak areas of the detected features were subsequently generated, followed by data normalization using MetaboAnalyst 5.0 software. Partial least squares-discriminant analysis (**PLS-DA**), differential metabolites analysis (with screening criteria of fold change (**FC**) ≥ 2 or ≤ 0.5, and *P* < 0.05), and variable importance in projection (VIP > 1.0) were implemented via the MetaboAnalyst online platform (https://www.metaboanalyst.ca/, accessed on 25 June 2020) and R project. Differential metabolites that met the criteria of FC ≥ 2 or ≤ 0.5, *P* < 0.05, and VIP > 1.0 were annotated as follows: first, their exact mass-to charge ratio (m/z) data were matched against the Metlin and Human Metabolome Database databases to retrieve the corresponding accession numbers; subsequently, the metabolite identities were confirmed by alignment to the PubChem database. Finally, the functional enrichment analysis of the differential metabolites was performed using the Kyoto Encyclopedia of Genes and Genomes (**KEGG**) database. The raw datas has been deposited in the China National Center for Bioinformation (CNCB) under accession number OMIX016368.ss

### Assessment of sperm quality and determination of fertility rate for semen incubated with L-arginine in vitro

We focused particularly on the arginine metabolic pathway, given its well-documented role in mammalian sperm function (Hemati et al., 2025; Li et al., 2025; Maciel et al., 2018), yet its specific involvement within the avian uterine fluid microenvironment remains poorly characterized.

Fresh semen collected from the 30 healthy roosters with proven sperm quality was thoroughly mixed and then equally divided into three groups. Each group included 3 replicates of 500 μL semen. The three groups were diluted at a ratio of 1:1 with PBS supplemented with 0.143 mM l-arginine (A0013-25 g, Solarbio, China), PBS supplemented with 0.287 mM l-arginine, and PBS (as a control), respectively. They were stored at 4°C for the assessment of the sperm motility and other parameters as above at 0, 2, 4, 6, and 8 h, respectively.

For the AI, a total of 150 experimental hens were selected randomly. The AI time was uniformly arranged at 2 *p.m*. At this time, most hens had completed laying eggs (there were no hard-shelled eggs in the uterus). Hens were divided into three groups (n = 50/group) and were subjected to a single AI directly into the upper vagina 2-4 cm around the UVJ, with a pipette used for precise quantitative insemination with 3 × 10⁹ cells/mL sperm in a 30 μL dose of diluted semen per hen. The eggs collected for 10 days after AI with date marked were set for incubation at the same in the same incubator following the standard incubation procedure. The eggs were candled at 10th days post incubation to check for fertility of hens in each group.

### Statistical analysis

All data were assessed for normality and homogeneity of variance using GraphPad Prism 9.5.0. For datasets conforming to normal distribution, one-way ANOVA followed by Tukey's multiple comparisons test was employed. Non-normally distributed datasets were analyzed using nonparametric Kruskal-Wallis analysis with Dunn's post hoc multiple comparisons test to determine significant differences in sperm motility, sperm kinetics parameters, 10-day fertility rate, and the average number of fertilized eggs.

The fertility data was fitted based on the following logistic functions

$$y=\alpha /\left(1+e^{-\beta \tau -x}\right)$$

where, y = fertility rate, x = days after the last insemination, α = theoretical maximum fertility rate, β = parameter of the rate of change in fertility rate, τ = days when the fertility rate is half of the maximum fertility rate. The parameters were estimated by iterative least squares (Kirby and Froman, 1990).

## Results

### *The effect of UF on sperm quality in vitro*

We statistically analyzed sperm kinetics parameters after incubation with PBS, PF and CF at 4°C for 0, 2, 4, 6 h, and 8 h under *in vitro* conditions (Table 1, Supplementary Table 2-1). At 0 h, sperm motility in the PF group was the highest at 77.53 ± 2.42%, which was higher than that in the PBS group (41.43 ± 5.88%) and the CF group (65.23 ± 1.88%) (*P* < 0.05). As time progressed, sperm motility in the PF group reached 83.47 ± 5.42% at 2 h, 82.33 ± 1.48% at 4 h, 90.10 ± 3.68% at 6 h, and 87.83 ± 0.67% at 8 h, consistently remaining significantly higher than those in the PBS (*P* < 0.05) and CF groups. The PBS group had the lowest sperm motility.

**Table 1:** Sperm kinetics parameters of sperm incubated with uterine fluid for different time at 4°C *in vitro*.

Incubation time	Sperm Motility (%)			VSL (μm/s)			VCL (μm/s)			VAP (μm/s)			ALH (μm)		
	PBS	PF	CF	PBS	PF	CF	PBS	PF	CF	PBS	PF	CF	PBS	PF	CF
0 h	41.43 ± 5.88^b^	77.53 ± 2.42^a^	65.23 ± 1.88^ab^	22.53 ± 2.23^b^	74.91 ± 3.44^a^	45.80 ± 4.00^ab^	59.94 ± 3.83^b^	134.49 ± 6.79^a^	117.90 ± 1.79^a^	31.81 ± 2.11^b^	92.44 ± 1.85^a^	67.47 ± 4.79^ab^	4.10 ± 0.14	5.76 ± 0.58	5.84 ± 0.20
2 h	33.23 ± 3.63^b^	83.47 ± 5.42^a^	71.37 ± 7.25^a^	15.26 ± 1.87^b^	42.96 ± 10.12^a^	32.60 ± 3.26^ab^	52.54 ± 2.63^b^	99.52 ± 5.09^a^	89.23 ± 5.04^a^	26.15 ± 1.30	57.86 ± 8.27	48.19 ± 3.48	4.88 ± 0.50	5.87 ± 0.55	5.29 ± 0.29
4 h	45.77 ± 3.34^b^	82.33 ± 1.48^a^	79.00 ± 5.20^a^	25.09 ± 1.47	35.50 ± 12.70	44.66 ± 17.49	80.70 ± 17.04	89.05 ± 15.11	83.10 ± 15.56	38.76 ± 6.03	49.77 ± 11.79	53.13 ± 16.84	5.65 ± 0.91	5.86 ± 1.25	4.30 ± 0.60
6 h	41.00 ± 2.49^b^	90.10 ± 3.68^a^	75.10 ± 4.16^a^	31.29 ± 7.68	38.53 ± 10.80	27.63 ± 3.63	82.30 ± 19.78	85.83 ± 12.94	62.03 ± 2.80	44.31 ± 10.52	50.04 ± 10.95	36.18 ± 2.85	5.20 ± 0.90	5.29 ± 0.91	3.58 ± 0.23
8 h	32.07 ± 0.59^b^	87.83 ± 0.67^a^	68.63 ± 10.11^a^	21.25 ± 2.90	33.30 ± 10.80	33.62 ± 13.58	62.19 ± 11.60	57.72 ± 6.99	64.15 ± 12.08	32.13 ± 3.51	38.69 ± 10.02	39.76 ± 12.86	4.15 ± 1.41	3.45 ± 0.41	3.54 ± 0.57

PBS, phosphate-buffered saline; PF, plumping fluid; CF, calcifying fluid; VCL, curvilinear velocity; VSL, straight-line velocity; VAP, average path velocity; ALH, amplitude of lateral head displacement. Data are shown by mean ± SEM. a-b, Mean values with no common superscripts within each row differ significantly (*P* < 0.05).

At 0 h, the VSL of the PF group was 74.91 ± 3.44 μm/s, higher than that of the PBS (22.5 ± 2.23 μm/s) and CF group (45.8 ± 4.00 μm/s) (*P* < 0.05). At 2 h, the VSL of the PF, CF, and PBS group decreased to 42.96 ± 10.12, 32.60 ± 3.26, and 15.26 ± 1.87 μm/s. With further increase in time, the VSL of all three groups decreased, but the PF group still maintained a relative high level at each time point. This trend was similar for other parameters including VAP, VCL. As for ALH, there were no statistically significant differences among groups.

### *Metabolome analysis of uterine fluid*

**Fig. 2:**
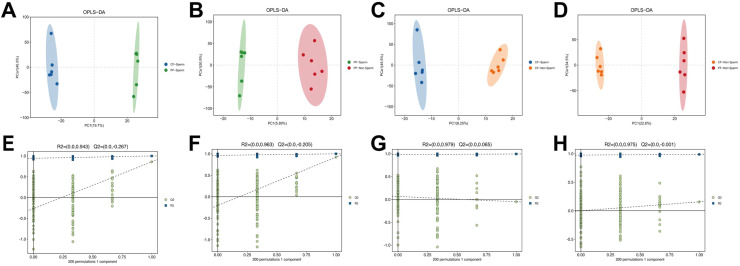
OPLS-DA model sequencing verification diagram. OPLS-DA model score between PF-Sperm and CF-Sperm (A), between PF-Non sperm and CF-Non sperm (B), between PF-Sperm and PF-Non sperm(C), and between CF-Sperm and CF-Non sperm (D). OPLS-DA model’ s permutation tests between PF-Sperm and CF-Sperm (E), between PF-Non sperm and CF-Non sperm (F), between PF-Sperm and PF-Non sperm (G), between CF-Sperm and CF-Non sperm (H). Note: R2 and Q2 represented the interpretation rate of the model to the matrix and the prediction ability of the model. The closer the value was to 1, the more stable and reliable the model was. The horizontal line corresponds to R2 and Q2 of the original model. Green and blue dots represented R2 and Q2 of the model after Y was replaced.

Orthogonal partial least squares structural discriminant analysis (**OPLS-DA**) score plots combined with permutation test models were employed to visualize and validate intergroup differences in metabolite profiles (Fig. 2). The OPLS-DA score plot exhibited a distinct separation between PF-Sperm and CF-Sperm (R2X=0.408, Q2=0.927), indicating that the model was reliable with good predictive ability (Fig. 2A). Similar clear discrimination was also observed between PF-Non sperm and CF-Non sperm groups (R2X=0.399, Q2=0.907) and between CF-Sperm and CF-Non sperm groups (R2X=0.397, Q2=0.707) (Fig. 2B and C). By contrast, an overfitting phenomenon was noted in the OPLS-DA model corresponding to the PF-Sperm and PF-Non sperm groups (Fig. 2D).

**Fig. 3:**
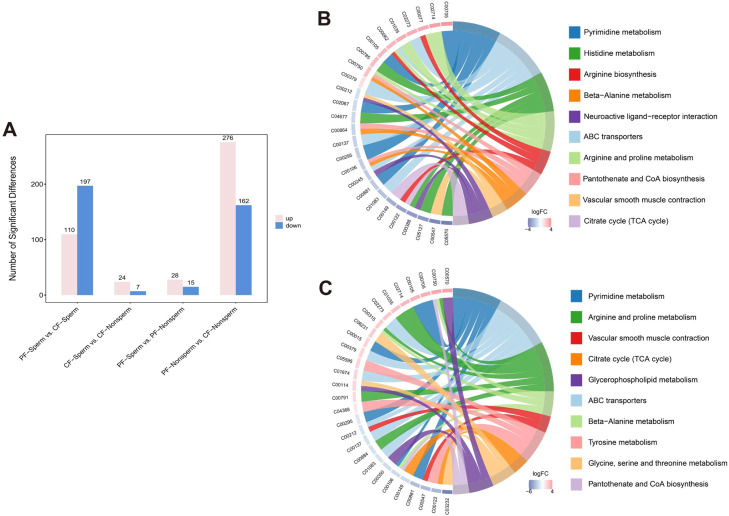
(A) Bar chart of the number of differential metabolites between PF-Sperm and CF-Sperm, between CF-Sperm and CF-Non sperm, between PF-Sperm and PF-Non sperm, and between PF-Non sperm and CF-Non sperm. Chord diagram of KEGG enrichment analysis of differential metabolites between (B) PF-Sperm and CF-Sperm, (C) between PF-Non sperm and CF-Non sperm. Note: The right panel displays the top 10 metabolic pathways with the highest significance. The left panel illustrates the differential metabolites within each pathway, where red and blue denote up-regulated and down-regulated metabolites, respectively. Color intensity corresponds to the magnitude of fold change (FC) for each metabolite.

Through non-targeted metabolomic, we identified 2,867 putative metabolites in the UF of laying hens (Supplementary Table 3). Firstly, to characterize the dynamic changes in metabolites between the paired groups of PF-Sperm vs. CF-Sperm and PF-Non sperm vs. CF-Non sperm, univariate t-test analysis was performed based on the metabolites fold changes between the two sets of comparison. The PF-Sperm vs. CF-Sperm comparison identified 110 up-regulated and 197 down-regulated metabolites (accounting for 10.7% of the total), whereas the PF-Non sperm vs. CF-Non sperm comparison detected 276 up-regulated and 162 down-regulated metabolites (accounting for 15.2% of the total). In addition, we specially investigated the effect of sperm presence on UF metabolites alternation, with a focus on the differential metabolites between the paired groups of PF-Sperm vs. PF-Non sperm and CF-Sperm vs. CF-Non sperm. The PF-Sperm vs. PF-Non sperm comparison identified 28 up-regulated and 15 down-regulated metabolites (accounting for 1.4% of the total), whereas the CF-Sperm vs. CF-Non sperm comparison detected 24 up-regulated and 7 down-regulated metabolites (accounting for 1.1% of the total) (Fig. 3A).

The KEGG pathway enrichment analysis of the differential metabolites indicated 7 pathways overlapped among the top 10 enriched pathways between PF-Sperm vs. CF-Sperm and PF-Non sperm vs. CF-Non sperm. These shared pathways included Pyrimidine metabolism, ABC transporters, Arginine and proline metabolism, beta-Alanine metabolism, Vascular smooth muscle contraction, Citrate cycle, and Pantothenate and CoA biosynthesis (Fig. 3B).

Table 2, Table 3 show the differential metabolites under the top 10 pathways in PF-Sperm vs. CF-Sperm and PF-Non sperm vs. CF-Non sperm respectively. There are a total of 16 identical differential metabolites, including Spermine, Malic acid, and N-Acetylputrescine. These metabolites are mainly related to the arginine metabolism pathway, either arginine itself or the intermediate products related to arginine metabolism, such as spermine and spermidine. In addition, there are also multiple metabolites related to lipid metabolism, such as CDP-ethanolamine and D-Pantothenic acid, and those related to antioxidation, such as ergothioneine and homocarnosine.

**Table 2:** The differential metabolites within the top 10 metabolic pathways with the highest significance between PF-Sperm vs. CF-Sperm.

ID	Metabolites	VIP	FoldChange	Regulation	*p*-value
C00705	dCDP	2.57	13.65	Up	<0.01
C02714	N-Acetylputrescine	2.37	11.11	Up	<0.01
C00077	L—Ornithine	2.10	8.07	Up	0.01
C02273	4-O-alpha-d-Galactopyranuronosyl-d-galacturonic acid	1.94	7.95	Up	0.04
C01035	4-Guanidinobutanoic acid	2.09	7.83	Up	0.01
C00062	L-Arginine	1.90	6.17	Up	0.02
C00105	Uridine 5′-monophosphate	2.04	6.14	Up	0.01
C00785	Urocanic acid	1.81	5.46	Up	0.03
C00750	Spermine	1.69	4.62	Up	0.04
C00379	Xylitol	1.34	2.43	Up	0.02
C00212	Adenosine	1.47	0.46	Down	<0.01
C02067	Pseudouridine	1.32	0.40	Down	0.02
C04677	AICAR	1.37	0.37	Down	0.03
C00864	D-Pantothenic acid	1.70	0.33	Down	0.00
C00137	Inositol	1.77	0.33	Down	<0.01
C00295	Orotic acid	1.82	0.30	Down	<0.01
C00106	Uracil	1.86	0.29	Down	<0.01
C00245	Taurine	1.68	0.29	Down	<0.01
C00881	2′-Deoxycytidine	1.58	0.27	Down	0.03
C01083	D-(+)-Trehalose	2.06	0.23	Down	<0.01
C00149	Malic acid	2.35	0.14	Down	<0.01
C00122	Fumaric acid	2.51	0.10	Down	<0.01
C00388	Histamine	2.22	0.09	Down	0.02
C05127	1-Methylhistamine	2.25	0.08	Down	0.02
C00547	Norepinephrine	2.87	0.05	Down	<0.01
C05570	Ergothioneine	3.00	0.04	Down	<0.01

**Table 3:** The differential metabolites within the top 10 metabolic pathways with the highest significance between PF-Non sperm vs. CF-Non sperm.

ID	Metabolites	VIP	FoldChange	Regulation	*p*-value
C00570	CDP-ethanolamine	2.26	17.39	Up	0.04
C00750	Spermine	2.41	16.43	Up	0.01
C00705	dCDP	2.42	11.91	Up	<0.01
C00105	Uridine 5′-monophosphate	2.14	10.62	Up	0.03
C02714	N-Acetylputrescine	2.41	8.71	Up	<0.01
C01035	4-Guanidinobutanoic acid	2.07	6.72	Up	0.01
C02273	4-O-alpha-d-Galactopyranuronosyl-d-galacturonic acid	1.95	5.78	Up	0.01
C00315	Spermidine	1.84	5.09	Up	0.01
C06231	Ectoine	1.96	4.91	Up	0.00
C00015	Uridine 5′-diphosphate	1.57	4.00	Up	0.04
C00379	Xylitol	1.89	3.79	Up	0.00
C05595	4-Hydroxyphenylacetylglutamic acid	1.59	3.77	Up	0.03
C01674	N,N'-diacetylchitobiose	1.58	3.74	Up	0.04
C00114	Choline	1.84	3.70	Up	<0.01
C00791	Creatinine	1.72	3.10	Up	<0.01
C04368	Beta-Tyrosine	1.24	2.15	Up	0.02
C00295	Orotic acid	1.37	0.48	Down	<0.01
C00212	Adenosine	1.54	0.40	Down	<0.01
C00137	myo-Inositol	1.45	0.44	Down	<0.01
C00884	Homocarnosine	1.58	0.39	Down	<0.01
C01083	D-(+)-Trehalose	1.59	0.39	Down	<0.01
C00350	PE(P-16:0/22:6)	1.04	0.31	Down	0.32
C00106	Uracil	1.78	0.32	Down	<0.01
C00149	Malic acid	1.51	0.32	Down	0.03
C00881	2′-Deoxycytidine	2.20	0.14	Down	<0.01
C00547	Norepinephrine	2.47	0.13	Down	<0.01
C00122	Fumaric acid	2.49	0.12	Down	<0.01
C03232	Phosphohydroxypyruvic acid	3.42	0.02	Down	<0.01

**Fig. 4:**
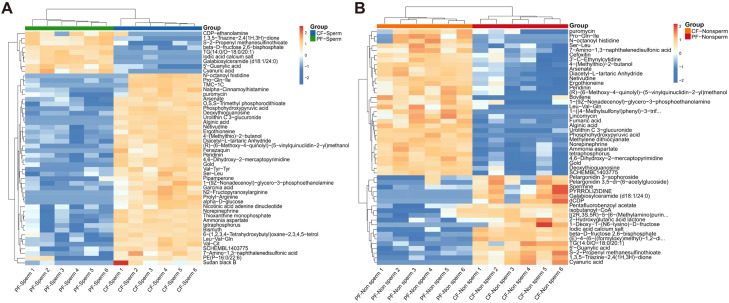
(A) Heat maps of top 50 most significantly differential metabolite between PF-Sperm and CF-Sperm, (B) between PF-Non sperm and CF-Non sperm. Note: Red represented increased metabolites, blue represented decreased metabolites.

Fig. 4B indicated that 32 differential metabolites overlapped among the top 50 differential metabolites of the PF-Sperm vs. CF-Sperm and PF-Non sperm vs. CF-Non sperm comparison groups. These overlapping metabolites were primarily associated with lipid metabolism (e.g., Galabiosylceramide (d18:1/24:0), TG (14:0/O-18:0/20:1), PE(P-16:0/22:6)), and antioxidant processes (e.g., Ergothioneine,Urolithin C 3-glucuronide, Peridinin). Furthermore, among the top 50 differential metabolites, several metabolites directly or indirectly associated with arginine metabolism were identified. Notably, compounds directly containing arginine residues, such as N2-Fructopyranosylarginine and Prolyl-Arginine, were observed. Key metabolites in the arginine metabolic pathway, including Ammonia aspartate and spermine, were also significantly altered.

As illustrated in Supplementary Table 4, the differentially metabolites identified in both the PF-Sperm vs. PF-Non sperm and CF-Sperm vs. CF-Non sperm comparisons were predominantly lipid and fatty acid metabolites (21 out of 43 and 13 out of 31, respectively), such as 7.61-fold up-regulated 2-Ethylpropanedioylcarnitine, 4.04-fold up-regulated 17-phenyl-13E-heptadecenoic acid and 6.37-fold up-regulated Cer(m18:1/24:1) in PF-Sperm vs. PF-Non sperm group, 3.51-fold up-regulated 21-oxo-docosanoic acid, 2.35-fold up-regulated 21‑hydroxy-heneicosanoic acid, and 2.12-fold up-regulated 11‑methoxy-nonadecanoic acid in CF-Sperm vs. CF-Non sperm group. Notably, organic acids, particularly amino acid-related metabolites, also exhibited significant alterations, including 3.45-fold up-regulated N—Oleoyl glutamine and 2.21-fold up-regulated N-Lauroylsarcosine in the PF-Sperm vs. PF-Non sperm, and 2.80-fold up-regulated Prolyl-Arginine along with 2.79-fold up-regulated Leu-Val-Gln in the CF-Sperm vs. CF-Non sperm. In the comparison between PF-Sperm and PF-Non sperm, we also identified multiple antioxidants-related metabolites, such as 16-Nitroxystearate and 1,5-Isoquinolinediol.

### *The influence of alterations in metabolites and metabolic pathways on sperm storage*

Metabolomic profiling of UF metabolites at different time points revealed distinct alterations in both metabolites and metabolic pathways. We observed significant differences in arginine-related metabolites across different groups (Table 2, Table 3; Fig. 4). Pathways involved in arginine biosynthesis and arginine and proline metabolism were notably enriched in in both the PF-Sperm vs. CF-Sperm, and PF-Non sperm vs. CF-Non sperm comparison (Fig. 3C). Moreover, L-arginine exhibited a relatively high FC (6.17) in PF-Sperm vs. CF-Sperm (Table 2). To validate the role of arginine on sperm storage within the SSTs, varying concentrations of L-arginine were supplemented into PBS for sperm incubation *in vitro*. Sperm motility was subsequently assessed at 0, 2, 4, 6, and 8 hours post incubation to evaluate the effects of arginine. Furthermore, AI was directly performed using the sperm incubated for 6 hours to determine the effect of L-arginine on fertility rate.

We statistically analyzed the sperm kinetics parameters of samples incubated *in vitro* at 4°C for 0, 2, 4, 6, and 8 h in three distinct media: PBS, PBS supplemented with 0.143 mM l-arginine, and PBS supplemented with 0.287 mM l-arginine (Table 4, Supplementary Table 2-2). At the 0 h time point, the sperm motility in the PBS control group was 84.03 ± 2.41%,with the 0.143 mM and 0.287 mM groups reaching 77.37 ± 1.44% and 85.70 ± 2.17%, respectively. After 4 h of incubation, sperm motility in the PBS group increased slightly to 58.83 ± 1.71%, whereas the 0.143 mM and 0.287 mM l-arginine groups exhibited higher motility values of 78.33 ± 7.74% and 74.93 ± 4.41%, respectively. However, by 6 h of incubation, sperm motility in the PBS decreased to 26.9 ± 5.19%, while the 0.143 mM and 0.287 mM l-arginine groups still maintained relatively high motility at 50.73 ± 2.06% and 70.03 ± 2.60%, respectively. At the 8 h time point, sperm motility in the PBS group dropped to only 18.87 ± 6.14%, in contrast to 52.93 ± 8.08% in the 0.143 mM group and 53.70 ± 1.20% in the 0.287 mM l-arginine group. Statistical analysis showed significant differences in sperm motility among the three groups across 4, 6, 8 h incubation time points (*P* < 0.05), suggesting that l-arginine supplementation can effectively enhance and sustain sperm motility during *in vitro* storage.

**Table 4:** Sperm kinetics parameters of sperm incubated with different concentrations of l-arginine for different time at 4°C *in vitro.*

Incubation time	Sperm Motility (%)			VSL (μm/s)			VCL (μm/s)			VAP (μm/s)			ALH (μm)		
	PBS	0.143 mM	0.287 mM	PBS	0.143 mM	0.287 mM	PBS	0.143 mM	0.287 mM	PBS	0.143 mM	0.287 mM	PBS	0.143 mM	0.287 mM
0 h	84.03 ± 2.41	77.37 ± 1.44	85.70 ± 2.17	48.80 ± 4.52	38.35 ± 1.53	53.32 ± 10.32	128.24 ± 6.48	125.55 ± 3.17	118.57 ± 9.62	73.02 ± 7.19	63.40 ± 1.23	73.75 ± 8.73	5.92 ± 0.23	6.25 ± 0.13	6.36 ± 0.39
2 h	66.45 ± 1.15	79.80 ± 3.91	78.97 ± 3.92	40.55 ± 1.33	52.53 ± 5.87	42.15 ± 2.53	109.02 ± 15.24	125.28 ± 5.39^a^	92.42 ± 6.53^b^	61.48 ± 6.59	73.29 ± 6.77^a^	55.41 ± 0.89^b^	5.71 ± 0.79	6.37 ± 0.21	5.35 ± 0.79
4 h	58.83 ± 1.71^b^	78.33 ± 7.74^a^	74.93 ± 4.41^a^	28.97 ± 0.47	30.82 ± 8.11	32.78 ± 1.52	97.99 ± 8.29	84.88 ± 8.70	96.83 ± 3.76	48.24 ± 2.35	45.19 ± 9.17	51.00 ± 2.17	5.70 ± 0.49	5.09 ± 0.24	5.96 ± 0.05
6 h	26.9 ± 5.19^c^	50.73 ± 2.06^b^	70.03 ± 2.60^a^	27.69 ± 4.10	35.67 ± 1.01	40.65 ± 2.03	93.59 ± 2.90	90.05 ± 2.47	86.37 ± 8.02	44.24 ± 3.06	47.58 ± 2.21	52.76 ± 3.92	4.94 ± 0.37	4.25 ± 0.13	5.30 ± 0.45
8 h	18.87 ± 6.14^b^	52.93 ± 8.08^a^	53.70 ± 1.20^a^	16.44 ± 1.48^b^	14.20 ± 2.21^b^	38.79 ± 10.35^a^	62.32 ± 5.10	55.04 ± 4.47	43.91 ± 17.52	27.81 ± 0.58	25.36 ± 2.00	33.60 ± 6.48	3.33 ± 0.33	4.06 ± 0.36	2.59 ± 0.91

PBS, phosphate-buffered saline; 0.143 mM, PBS supplemented with 0.143 mM l-arginine; 0.287 mM, PBS supplemented with 0.287 mM l-arginine; VCL, curvilinear velocity; VSL, straight-line velocity; VAP, average path velocity; ALH, amplitude of lateral head displacement. Data are shown by mean ± SEM. a-c, Mean values with no common superscripts within each row differ significantly (*P* < 0.05).

As incubation time progressed, VSL, VCL, VAP, and ALH showed a declining trend across all groups. At 8 h of incubation, the VSL in the 0.287 mM L-arginine was significantly greater than that in the PBS control group (*P* < 0.05), while no significant differences were noted at other time points (*P* > 0.05). At 2 h of incubation, the VCL and VAP in the 0.287 mM l-arginine were significantly higher than those in the 0.143 mM l-arginine (*P* < 0.05), with no significant differences at the other time points (*P* > 0.05).

**Fig. 5:**
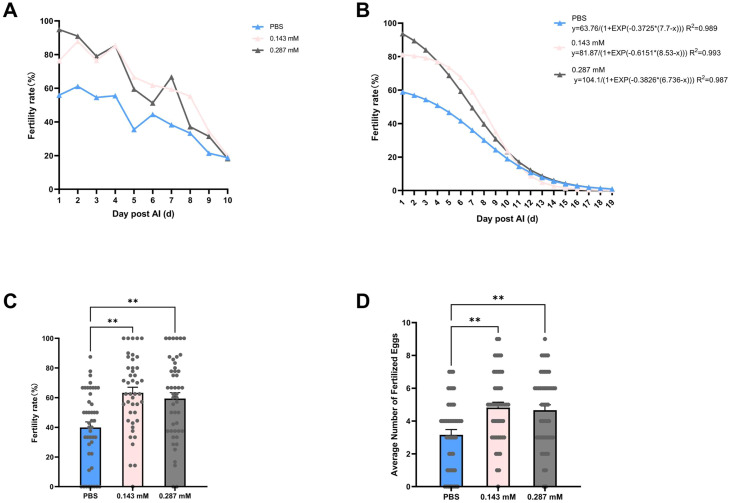
The effect of **L**-arginine supplementation on fertility duration. (A) Fertility rate over time. (B) The fitting curve of fertility rate over time. (C) The average 10-day fertility rate. (D) The average number of fertile eggs. Note: PBS, phosphate-buffered saline; 0.143 mM, PBS supplemented with 0.143mM l-arginine; 0.287 mM, PBS supplemented with 0.287 mM l-arginine. The error bars represented the standard error of the mean. ** Mean values within each column differed significantly (*P ≤ 0.01*).

As shown in Fig. 5A, the fertility rate was recorded within 10 days post AI, where insemination was performed using sperm samples incubated at 4°C for 6 hours in three media: PBS, PBS supplemented with 0.143 mM L-arginine (0.143 mM), and PBS supplemented with 0.287 mM l-arginine (0.287 mM). The fertility rate of the PBS group was significantly lower than that of the two l-arginine supplemented groups, with the 0.143 mM group exhibiting a marginally higher fertility rate than the 0.287 mM group. The fitted 19-day fertility curve, derived from 10-day data, further confirmed that l-arginine treatment prolongs sperm functional longevity in the hen reproductive tract (Fig. 5B).

Both l-arginine groups exhibited significantly higher fertility rates compared to the PBS group over a 10-day duration (*P* < 0.01) (Fig. 5C). The average number of fertile eggs was also significantly elevated in the l-arginine groups (*P* < 0.01) (Fig. 5D).

## Discussion

The UF in hens provides a critical microenvironment for sperm after their release from SSTs, with its composition shifts regularly between PF and CF phases during eggshell formation. Which UF supports greater sperm motility and storage efficiency, and the specific metabolites and pathways responsible remain unclear. This study employed non-targeted metabolomics to compare the metabolite profiles of PF and CF from inseminated and non-inseminated hens, aiming to identify key metabolic regulators of sperm function for optimizing the reproduction efficiency.

Temperature and diluent qualities for *in vitro* spermatozoa storage are the most crucial criteria for the success of AI (Sarkar, 2020). Previous research results demonstrated a steady decline in the percentage of sperm motility during liquid storage of semen (Kheawkanha et al., 2025; Pardyak et al., 2024). The results here shows that PF and CF from hens has a marked fertility increasing action on spermatozoa exposed to it *in vitro,*with PF demonstrating a more pronounced effect than CF. This is consistent with previous studies that the uterine fluid of hens can prolong the survival time of sperm *in vitro* (Ahammad et al., 2013; Ahammad, 2013).

The present study revealed that sperm deposition by AI caused (within 24 h) changes in the metabolic composition of the avian UF, indicates that sperm have unique metabolic requirements. This is in line with previous studies which demonstrated that sperm deposition after natural mating induced rapid (within 24 h) changes of gene expression in the uterus and SSTs (Atikuzzaman et al., 2015) and proteomic composition in UF (Riou et al., 2019). The metabolomic differences between PF and CF, indicate distinct metabolic microenvironments in the plumping and calcifying phases. The presence or absence of sperm further modulates these metabolic profiles.

Lipid metabolism, particularly fatty acid metabolism, may actively participate in spermatozoa energy metabolism and motility regulation (Asghari et al., 2017; Paiva et al., 2015; Rato et al., 2012; Samadian et al., 2010). Sperm stored within the SSTs utilized metabolized lipids, primarily fatty acids, to prolong their viability and survival duration, and that the viability of sperm cultured in medium containing oleic acid and linoleic acid was higher than that of the control (Huang et al., 2016). Previous research demonstrated extensive lipid distribution within UVJ tissues containing SSTs, and revealed significant enrichment of unsaturated fatty acid metabolism pathways by the serum metabolism of hens exhibiting high versus low sperm storage capacity (Yang et al., 2021). We observed differential expression of multiple lipid and fatty acid metabolites in the UF of PF-Sperm vs. CF-Sperm, PF-Non sperm vs. CF-Non sperm. This metabolic profile suggests that these lipids and fatty acids potentially serve as energy substrates during sperm ascension and contribute to sperm maintenance mechanisms.

Oxidative damage is an important factor contributing to sperm mortality during semen storage. Both sperm and seminal plasma contain substances with inherent antioxidant capabilities (Partyka and Nizanski, 2021). In the present study, multiple antioxidant metabolites were identified among the differential metabolites in both the PF-Sperm vs. CF-Sperm and PF-Non sperm vs. CF-Non sperm comparisons. The presence of these antioxidant metabolites may play a role in maintaining sperm viability.

In our study, arginine associated metabolites were significantly up-regulated in PF, and arginine related pathways were significantly enriched pathways in both PF and CF groups. l-arginine actively participates in sperm formation and has a protective effect against lipid peroxidation on the sperm (Srivastava et al., 2000). A deficiency in l-arginine causes derangement of sperm metabolism leading to decrease in motility and loss of spermatogenesis (Hassanpour et al., 2010; Srivastava et al., 2006). It has been revealed that l-arginine plays an important role in stimulating sperm motility in humans, goats, boars, and bulls under *in vitro* conditions (Aydin et al., 1995; Ren et al., 2015; Srivastava et al., 2006). Administration of l-arginine to oligospermic and asthenospermic patients results in an improvement in both sperm count and motility without any side effects (Aydin et al., 1995). Previous studies have highlighted the impact of arginine concentration on sperm parameters. For example, compared to the control group, l-arginine at lower concentrations (10 mM or below) not only maintained the sperm motility but also promoted sperm capacitation (O'Flaherty et al., 2004). They further indicated that l-arginine exerted no adverse effects on cell viability throughout the experiment period. *In vitro* experimental results have also revealed l-arginine supplementation enhances boar sperm capacitation and acrosome reaction (Chen et al., 2018; Funahashi, 2002). Moreover, dietary supplementation with l-arginine has been shown to exert positive effects on reproductive performance and semen quality in roosters (Ahangar et al., 2017). The addition of l-arginine at different concentrations to the PBS significantly prolonged the *in vitro* sperm quality. Subsequent AI performed using the semen incubated with l-arginine-supplemented PBS for 6 hours resulted in a fertility rate substantially higher than that incubated with PBS control group. These results confirm the efficacy of arginine in sustaining sperm viability and reproductive competence.

Given that our *in vitro* experiments were conducted at 4°C, which does not fully recapitulate the physiological temperature of the hen oviduct (approximately 41°C), future studies should validate the key findings under more physiological conditions. Furthermore, while our metabolomics analysis identified multiple enriched pathways (e.g., lipid metabolism), their causal roles in regulating sperm motility remain to be established.

### Conclusions

UF metabolic profiles differ significantly between plumping and calcifying phases, with sperm presence inducing dynamic metabolic adjustments. PF exhibits superior efficacy in maintaining sperm motility and kinetics parameters compared to CF. Key pathways including TCA cycle, lipid metabolism, and arginine metabolism synergistically regulate sperm energy supply, survival, and function. The supplementation of l-arginine, which was a key up-regulated metabolism in the PF, enhances rooster sperm motility, kinetics parameters, and fertility rate. These findings provide a theoretical basis for optimizing poultry AI and sperm storage techniques for improving reproductive efficiency.

## CRediT authorship contribution statement

**Qi Zhang:** Writing – review & editing, Writing – original draft, Conceptualization. **Hongfeng Du:** Formal analysis. **Caiyue Ge:** Data curation. **Zhouying Zhang:** Software. **Jingwei Yuan:** Validation, Software. **Yunlei Li:** Validation. **Adamu Mani Isa:** Data curation. **Jilan Chen:** Supervision. **Yanyan Sun:** Writing – review & editing, Supervision, Conceptualization.

## Disclosures

All authors have no commercial or financial conflicts of interest related to this study, and the research process was not interfered with by any interested parties.

## Appendix Supplementary materials

Image, application 1

Image, application 2

Image, application 3

Image, application 4

## Footnotes

Supplementary material associated with this article can be found, in the online version, at doi:10.1016/j.psj.2026.107102.
